# Effect of Alkyl Chain Length on Dissolution and Regeneration Behavior of Cotton in 1-Alkyl-3-methylimidazolium Acetate Ionic Liquids

**DOI:** 10.3390/molecules30132711

**Published:** 2025-06-24

**Authors:** Niwanthi Dissanayake, Vidura D. Thalangamaarachchige, Edward Quitevis, Noureddine Abidi

**Affiliations:** 1 Department of Chemistry and Physics, McNeese State University, Lake Charles, LA 70605, USA; ndissanayakeralalage@mcneese.edu; 2 Department of Chemistry and Biochemistry, Texas Tech University, Lubbock, TX 79409, USA; edward.quitevis@ttu.edu; 3 Fiber and Biopolymer Research Institute, Texas Tech University, Lubbock, TX 79403, USA

**Keywords:** cellulose, ionic liquids, cation, alkyl chain length, polarized light microscopy

## Abstract

Ionic liquids (ILs) have attained considerable attention as cellulose solvents. Nevertheless, the detailed mechanism of cellulose dissolution in ILs is not clearly defined. It is crucial to recognize the role of the individual components of the ILs to fully understand this mechanism. During this study, the effect of alkyl chain length in imidazolium cation was examined using synthesized ILs which are composed of common acetate anion and imidazolium cations with different alkyl substituents. This study also aimed to investigate the odd–even effect of alkyl chain carbons. Furthermore, whereas most published investigations on cellulose dissolution in ILs used microcrystalline cellulose (MCC), which has a far lower degree of polymerization, in this study, cotton cellulose was used. During the dissolution experiments, cotton cellulose (5% *w*/*w*) was added to each IL, and the progress of the dissolution was monitored using polarized light microscopy (PLM). The regeneration of cellulose was performed by using water as the anti-solvent, and the regenerated cellulose was characterized by Fourier-transform infrared (FTIR) spectroscopy and scanning electron microscopy (SEM). During these experiments, it was noted that ILs with odd C3 and C5 carbon chains were less effective at dissolving cellulose than those with even C2 and C4 alkyl chains. Additionally, after regeneration, biomaterials for a variety of applications could be produced.

## 1. Introduction

Cellulose is the main carbohydrate produced by plants in photosynthesis and accounts for approximately 40% of the raw plant materials of plants [1]. However, the secondary cell of a mature cotton fiber contains more than 99% cellulose [2]. Cellulose is a raw material for paper and textile industries and can also be converted into fibers, films, beads, and powders or can be transformed into functionalized materials by homogeneous derivatization [3,4,5]. Due to the hydrogen bonding network, cellulose is insoluble in water and other common organic solvents. Therefore, cellulose dissolution becomes a challenging task. A variety of solvent systems are employed to break down the hydrogen bonding network in cellulose. A few of these include N, N-dimethylacetamide/lithium chloride (DMAc/LiCl), sodium hydroxide-based solvent systems, N-Methylmorpholine N-oxide (NMMO), and aqueous inorganic salts [6,7,8]. However, there are several limitations that are associated with these solvents, such as toxicity, the requirement of a high temperature, lengthy dissolution times, complex dissolution processes, and the degradation of cellulose [9,10]. Most of these limitations can be minimized by utilizing ionic liquids as solvents for cellulose.

ILs are organic salts with an exceptional capacity to dissolve cellulose. They have many uses in electrochemistry, as electrolytes, in tribology and can be substituted for organic solvents. ILs are liquids at or below 100 °C and have a wide range of applications within electrochemistry and tribology, as well as being used as a replacement for organic solvents, battery electrolytes, gel electrolytes, etc. [11,12,13,14,15,16,17,18,19,20,21]. Task-specific ILs can be synthesized by tuning their anion and cation compositions [22].

The mechanism of cellulose dissolution in ILs was studied by examining the interactions between cellulose and the individual components of ILs. The solubility of cellulose is significantly impacted by the size and basicity of ILs’ anions, according to previous research [23,24,25,26,27,28,29]. Only a few studies have suggested that the cations of the ILs also play an important role in cellulose dissolution [5,30,31,32,33]. It was observed that imidazolium-based cations were found to be the most effective at dissolving cellulose among the heterocyclic cations. This is explained by the fact that the acidic protons in the imidazolium cation, in particular C-2 proton, are involved in the formation of hydrogen bonds between the cations and cellulose [32,34,35,36].

A study by Dissanayake et al. showed that the cotton cellulose dissolution was significantly impacted by the size and shape of the N-substituents in the imidazolium cation [30]. Another study by Ferreira and coworkers demonstrated that in an IL/DMSO binary mixture, the IL with alkyl chain (C_4_MeImAc) was more effective than the IL that has an alkoxy side chain (C_3_OMeImAc) on cellulose dissolution. This is due do lower basicity and higher viscosity of C_3_OMeImAc-DMSO to the higher viscosity, lower basicity, and the formation of weaker hydrogen bonds with cellulose than C_4_MeImAc/DMSO [37]. Moreover, the length of the alkyl chain of cations is an important factor which impacts the cellulose dissolution process [34,38,39,40,41,42,43]. According to some studies, ILs with short alkyl chains attached to imidazolium nitrogen heteroatoms are more effective in dissolving microcrystalline cellulose (MCC) than those with cations that have long alkyl chains [5,36,38,39,40,44,45,46].

Erdmenger and coworkers reported on the dissolution efficiency of MCC in 1-alkyl-3-methylimidazolium chloride ILs with different alkyl chains, C1 to C10, at 100 °C. Their results showed that cellulose solubility in these ILs did not decrease regularly with the increase in the length of alkyl chain of the ILs. Moreover, they found that ILs with short alkyl chains exhibited an odd even effect, whereby Ils with even numbered alkyl chains dissolved MCC more effectively than those with even number of alkyl chains [47]. Vitz et al. further extended the study and dissolved MCC in imidazolium-based chloride and bromide ILs with the length of the alkyl chains ranging from C1 to C10. They found that even-numbered chloride ILs with short alkyl chains had better dissolution ability than the other ILs used in their study. Furthermore, they also observed an odd–even effect only for 1-alkylimidazolium chloride ILs, but not for 1-alkylimidazolium bromide ILs [5]. In addition, Swatloski et al. also experimentally showed that 1-alkylimidazoluim chloride ILs with short alkyl chains ([C_4_C_1_im][Cl]) appeared to be more efficient in dissolving pulp than ILs with long alkyl chains (1-hexyl- or 1-octyl-3-methylimidazolium chloride) [39].

Several Molecular Dynamic Simulations (MD) were performed to investigate the effect of N- alkyl chain length of IL’s cations. Rabideau et al. examined the impact of steric hindrance caused by the length of the alkyl chains of ILs on cellulose dissolution [38]. In another study, Zhao et al. calculated the radial distribution function g(r) for the H atom and anion in an IL–cellulose system, the interaction energies, and the number of hydrogen bonds for 1-alkyl-3-methylimidazolium ILs, [C_n_C_1_im][Cl] (n = 2, 4, 6, 8,10). Their results showed that radial distribution function increased as the length of alkyl chains in the ILs’ cations increased. Moreover, the local density distribution of the anion (Cl^−^) around cellulose in the IL–cellulose system decreased with the increasing length of alkyl chains. The number of hydrogen bonds between cellulose and the IL cations at C2, C4, and C5 sites were greater for the ILs with cations that had shorter alkyl chains compared to those with longer chains [40].

Multiple cellulose dissolution studies were conducted using acetate-based ILs [42,48,49,50,51,52,53].

Kasprzak et al. examined the effect of the alkyl chain length of a series of substituted piperazinium, piperidinium, and pyrrolidinium acetates/DMSO on MCC dissolution at 25, 50, and 80 °C. Amongst all tested solvents, *N,N’*-dimethyl-*N*-ethylpiperazinium acetate ([DMEPpz][Ac]/DMSO) mixed solvent performed best in the solubility of cellulose. They found that cellulose dissolution in most IL/DMSO solvents decreased with increasing alkyl chain length [42]. Ren et al. utilized IL-DMSO mixtures with different ratios to investigate the cation alkyl chain length impact on cellulose dissolution. At higher DMSO/IL ratios, dissolution was enhanced for cations with longer alkyl chains whereas a 1:1 mixture was effective in cations with shorter alkyl chains [28]. Andanson et al. used 1-butyl-3-methylimidazolium acetate IL as a cellulose solvent. They found that 25 wt% of microcrystalline cellulose (MCC) was dissolved in this IL below 100 °C [48]. Clough and coworkers used acetate-based ILs and cellulose solubility was investigated. In their study, series of carbohydrates were dissolved in acetate-based ILs [49]. Zang at al synthesized 1,3-diallyl-2- ethyl imidazolium acetate IL to study cellulose solubility. Additionally, DMSO was used as a co-solvent [50]. Hawkins et al. used cellulosic flax fibers in the ionic liquid 1-ethyl-3-methylimidazolium acetate ([C2mim][OAc]) with the co-solvent DMSO in their study [51]. Anh le et al. developed cellulose solubility phase diagrams in two binary solvents based on 1-ethyl-3-methylimidazolium acetate (EmimAc) mixed with water and with DMSO. They found out that the solubility of cellulose in EmimAc–DMSO is significantly greater than that of EmimAc–water [52]. Muller et al. utilized 1-ethyl-3-methylimidazolium acetate to generate cellulose polymer blends. Cellulose was blended with various synthetic polymers using EmimAc and DMSO, leading to transparent blend films [53].

In the studies mentioned above, the focus was on investigating the effect of the alkyl chain length in halide-based ILs in cellulose dissolution. Nevertheless, no systemic study has been published for acetate-based ILs to date, and little is known about how the alkyl chain length of these ILs affects their solubility in cellulose. Moreover, the impact of alkyl chain length on a source of cellulose with a high degree of polymerization (DP), such as cotton cellulose, has not been studied either. There are no detailed systematic studies in the literature to investigate the even–odd effect with imidazolium-based acetate ILs in cotton cellulose dissolution.

The purpose of this work was to investigate how the length of the alkyl chains of 1-alkyl-3-methylimidazolium acetate-based ILs affects the dissolution of cotton cellulose. Seven ILs were synthesized with alkyl chains ranging from C2 to C8 on the imidazolium cations ([C_n_C_1_im]^+^; n = 2, 3, 4, 5, 6, 7, 8), and these cations were combined with common acetate anion (See Table 1). In each IL, 5% *w*/*w* cotton cellulose was dissolved at 90 °C (Figure S1). The extent of dissolution of cotton cellulose in each IL was monitored using polarized light microscopy. After the dissolution, cellulose was regenerated from each cellulose/IL solution, and the surface morphology of the regenerated cellulose was examined by scanning electron microscopy (SEM) and Fourier-transform infrared (FTIR) spectroscopy.

## 2. Results

### 2.1. Polarized Light Microscopy of Cellulose Dissolution

**Figure 1:**
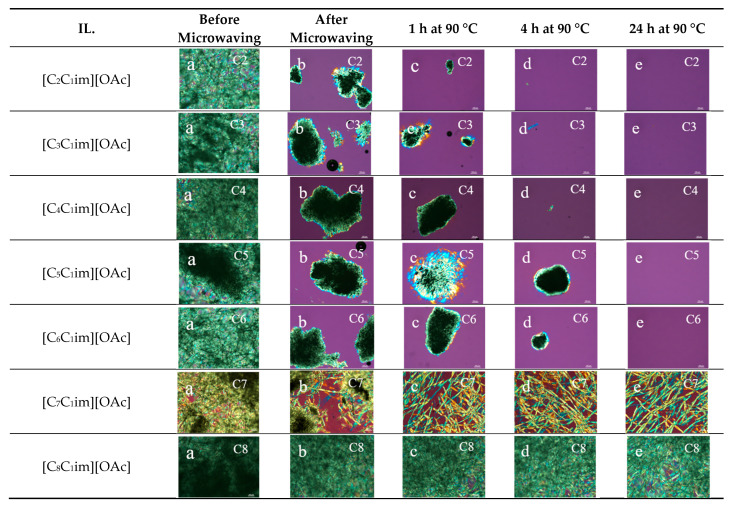
PLM images of cotton dissolved in [C_2_C_1_im][OAc], [C_3_C_1_im][OAc], [C_4_C_1_im][OAc], [C_5_C_1_im][OAc], [C_6_C_1_im][OAc], [C_7_C_1_im][OAc], and [C_8_C_1_im][OAc] at different time intervals (before and after microwaving, and after 1 h, 4 h, and 24 h at 90 °C).

The resulting PLM images, shown in Figure 1, reveal that the extent of dissolution changed depending on the length alkyl substituent in the IL. Initial microwaving facilitated the dissolution particularly in the ILs containing C2 (ethyl) to C6 (hexyl) carbons in the alkyl chains.

After 1 h at 90 °C, [C_2_C_1_im][OAc] was found to be the most effective IL at dissolving cotton cellulose compared to the others tested. However, a substantial amount of cotton cellulose was dissolved in other ILs including [C_3_C_1_im][OAc] and [C_4_C_1_im][OAc] at 4 h intervals. Further monitoring of the progress of the reaction indicated that [C_2_C_1_im][OAc] and [C_4_C_1_im][OAc] completely dissolved cotton cellulose within 8 h, whereas [C_3_C_1_im][OAc] showed a complete dissolution upon heating the mixtures 24 h at 90 °C (see Supporting Information). The PLM images show that [C_6_C_1_im][OAc] was more effective at dissolving cotton cellulose than [C_5_C_1_im][OAc] after 4 h of dissolution at 90 °C, which may be related to the odd–even effect. A complete dissolution of cotton fibers in [C_5_C_1_im][OAc] and [C_6_C_1_im][OAc] was achieved at 24 h. In contrast, [C_7_C_1_im][OAc] only caused the swelling of cotton fibers, and [C_8_C_1_im][OAc] showed no noticeable dissolution even after 24 h. Therefore, based on the PLM images, the effectiveness of the ILs in dissolving cellulose decreased in the following order: [C_2_C_1_im][OAc] > [C_4_C_1_im][OAc] > [C_3_C_1_im][OAc] > [C_6_C_1_im][OAc] > [C_5_C_1_im][OAc] > [C_7_C_1_im][OAc] > [C_8_C_1_im][OAc]. These results are consistent with findings from previous studies.

From the series of ILs, three ILs comprising short alkyl chains ([C_2_C_1_im][OAc], [C_3_C_1_im][OAc], and [C_4_C_1_im][OAc]) demonstrated a greater ability to dissolve cotton cellulose. In contrast, ILs with longer alkyl chains, particularly [C_7_C_1_im][OAc] and [C_8_C_1_im][OAc], were less effective in this regard, aligning with findings from multiple prior studies [32,33,36,41]. However, PLM image analysis revealed a contrasting result: [C_6_C_1_im][OAc] showed better solubility compared to [C_5_C_1_im][OAc]. This may be explained by the odd–even effect observed by Erdmenger et al. [45].

### 2.2. Characterization of Regenerated Cellulose

#### 2.2.1. Scanning Electron Microscopic Images of Regenerated Cellulose

**Figure 2:**
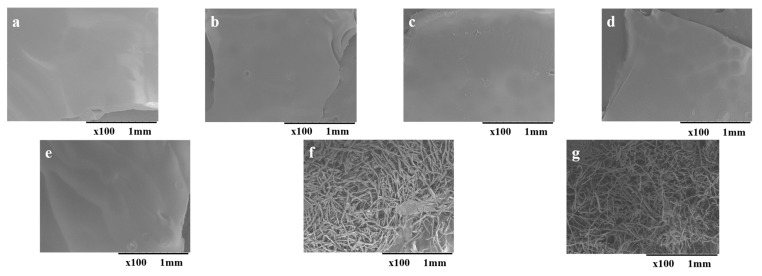
SEM images of cellulose regenerated after dissolving in (**a**) [C_2_C_1_im][OAc], (**b**) [C_3_C_1_im][OAc], (**c**) [C_4_C_1_im][OAc], (**d**) [C_5_C_1_im][OAc], (**e**) [C_6_C_1_im][OAc], (**f**) [C_7_C_1_im][OAc], and (**g**) [C_8_C_1_im][OAc] (×100).

The surface morphology of regenerated cellulose was analyzed using X100 scanning electron microscopy. The captured images were depicted in Figure 2. The regenerated cellulose obtained from [C_2_C_1_im][OAc], [C_3_C_1_im][OAc], [C_4_C_1_im][OAc], [C_5_C_1_im][OAc], and [C_6_C_1_im][OAc] showed uniform structures with no remaining undissolved fibers. This indicates that the native cotton cellulose completely dissolved in these ILs, which contained alkyl chains ranging from C2 to C6. However, cellulose regenerated from [C_7_C_1_im][OAc] and [C_8_C_1_im][OAc] solvents was predominantly composed of fibers that had not fully dissolved. This observation is consistent with PLM images, which also suggested the incomplete dissolution of cotton fibers in these ILs.

#### 2.2.2. FTIR Spectra of Regenerated Cellulose

**Figure 3:**
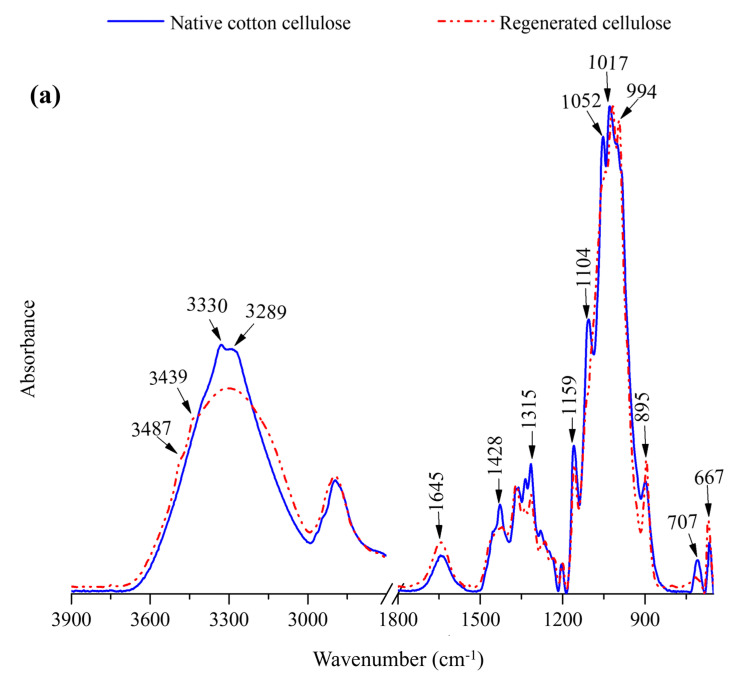

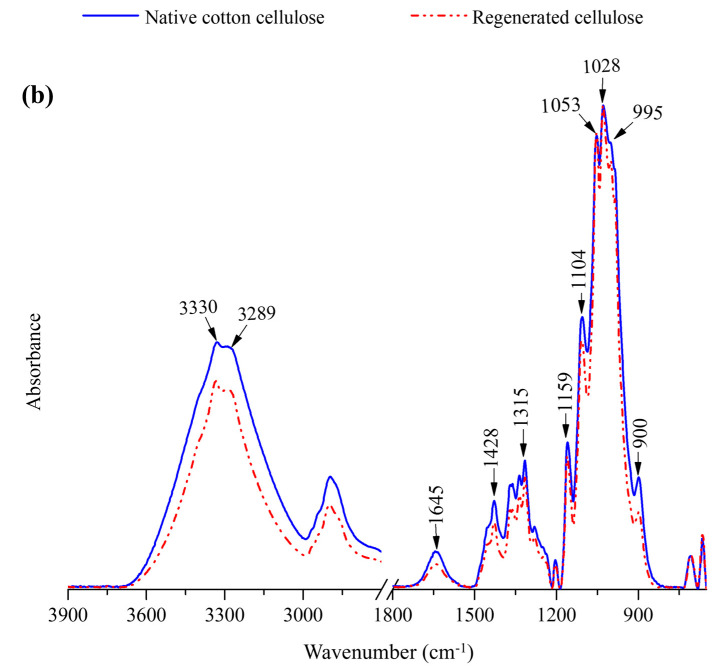
(**a**) FTIR spectra of hot-dried ground cotton fibers and cellulose regenerated after dissolving in [C_2_C_1_im][OAc]. (**b**) FTIR spectra of hot-dried ground cotton fibers and cellulose regenerated after dissolving in [C_8_C_1_im][OAc].

The FTIR spectra of native and regenerated cellulose materials from [C_2_C_1_im][OAc] and [C_8_C_1_im][OAc] are shown in Figure 3. (See Supporting information for the FTIR spectra of cellulose regenerated from [C_3_C_1_im][OAc], [C_4_C_1_im][OAc], [C_5_C_1_im][OAc], [C_6_C_1_im][OAc], and [C_7_C_1_im][OAc].) All the spectra are similar, and no new peaks are observed in regenerated cellulose samples, indicating that no chemical reaction occurred during the dissolution and regeneration processes. However, the FTIR spectra of cellulose regenerated after dissolution in ILs containing alkyl chain lengths C2−C6 showed few changes in the frequencies of the vibration bands at 3700–3000, 1428, 1315, 1159, 1104, 900, 707, and 667 cm^−1^ compared to the wavenumbers of the vibration bands of the original cotton cellulose. All these changes in the frequencies of the vibration bands of regenerated cellulose could be due to the transition of cellulose I of native cellulose into cellulose II during dissolution and regeneration processes. In contrast, the FTIR spectra of regenerated cellulose materials after dissolving in the least effective ILs ([C_7_C_1_im][OAc] and [C_8_C_1_im][OAc]) have wavenumbers in the vibration bands comparable to native cotton cellulose, indicating the retention of the crystalline I structure. In addition, the presence of new vibrations at 1730, 1578, and 1248 cm^−1^ in the [C_7_C_1_im][OAc]-regenerated cellulose suggested the fact that the IL was not completely removed during the regeneration process.

## 3. Discussion

The reduced ability of cellulose to dissolve in ILs with long alkyl chains is likely due to the increase in the viscosity of the cellulose/IL mixtures, which hinders the mass transport of ions [43,47,54,55]. Several studies also suggested that the length of the alkyl length has an influence on the distribution of the anion ([OAc-]) around the hydroxyl groups of cellulose [39,40]. Consequently, the lower solubility of cellulose in ILs [C_7_C_1_im][OAc] and [C_8_C_1_im][OAc] can be due to the reduced number of anions around cellulose. Another factor is that the longer alkyl chains on the cation have a strong steric effect that impedes interactions between the anion and cellulose. PMF (Potential of Mean Force) calculations reveal the energy barriers associated with cellulose chain separation, showing that the IL with short alkyl chains has the smallest energy barrier, while the IL with long alkyl chains has the largest [41]. Due to the steric hindrance and high PMF values, the ILs with longer alkyl chains obstruct or weaken the hydrogen bond formation between the IL and the linker oxygen of cellulose. This suggests that the number of hydrogen bonds formed between the ILs with longer alkyl chains and cellulose is reduced [40]. Furthermore, ILs with C7-hepty and C8-octyl alkyl chains are confronted with steric hindrance to intercalate between chains, leading to poor dissolution. Alkyl groups are generally electron-donating groups that stabilize the imidazolium nitrogen’s positive charge, particularly the acidity of the C2 hydrogen in the imidazolium cation, which helps form hydrogen bonds with cellulose and ultimately aids in the dissolution of cellulose. The capacity to donate electrons increases with the length of the alkyl chain. The acidity of the C2 hydrogen is often reduced by adding extra electron-donating groups to the imidazolium cation’s nitrogen atoms. This is because it is more difficult to extract the proton from the C2 hydrogen due to the higher electron density of nitrogen atoms. Longer alkyl chains in imidazolium cations reduce the acidity of the C2 hydrogen, resulting in weaker contact with cellulose and a tendency to decrease dissolution.

## 4. Materials and Methods

### 4.1. Cotton Cellulose

#### 4.1.1. Preparation of Cotton for Dissolution

Cotton slivers were received from the Fiber and Biopolymer Research Institute (FBRI), Texas Tech University. They were scoured and bleached using an established protocol. Then, cotton was ground using a Wiley Mill (Wiley Mini Mill 3383-L10: 115 V, 60 Hz, Thomas Scientific, Swedesboro, NJ, USA) and passed through a 20-mesh screen. The ground cotton fibers were dried at 105 °C in a laboratory oven overnight to remove the moisture.

#### 4.1.2. Dissolution of Ground Cotton Fibers in ILs

All acetate ILs are less viscous, highly hygroscopic liquids. First, ILs were weighed into clean and dry glass jars. Then, ground cotton fibers (5% *w*/*w*) were weighed separately. Cotton was gradually added to the ILs, and the mixtures were stirred by glass rods. Afterwards, the cotton/IL mixtures were heated in a microwave oven for 3–4 s pulses at full power until the temperature reached 90–100 °C. Later, the mixtures were transferred to a 90 °C laboratory oven and heated continuously for 24 h.

#### 4.1.3. Polarized Light Microscopy of Cellulose/IL Mixtures

A Nikon ECLIPSE LV 100 polarizing light microscope (Nikon Corporation, Tokyo, Japan) equipped with the NIS-Elements imaging platform was used to observe the progress of the dissolution of cotton cellulose in these ILs. The representative samples of the cellulose suspensions were collected at different time intervals: before and after the mixtures were heated in the microwave oven and after 1 h, 4 h, 8 h, and 24 h at 90 °C. Each mixture was stirred thoroughly before the samples were collected. Representative samples from each mixture were placed on clean, dry glass slides and covered using cover slips. The PLM images of these samples were recorded at room temperature under ×10 magnification.

#### 4.1.4. Regeneration of Cellulose from Each Cellulose/IL Mixture

Cellulose/IL mixtures were poured onto Teflon plates and covered with glass plates. Two plates were gently pressed to form uniform films of the mixtures. Teflon–glass plates that contained the cotton cellulose/IL mixtures were placed on glass pans. Cellulose from each mixture was precipitated by adding deionized water, and water was changed every 3–4 days to remove ILs from the regenerated cellulose.

#### 4.1.5. Drying the Regenerated Cellulose

Regenerated cellulose samples from each mixture were transferred to plastic containers and kept at −4 °C for 2 h. Then, the regenerated cellulose samples were freeze-dried using a Labconco FreeZone (4.5 Liter Cascade Benchtop Freeze Dry System, Labconco Corporation, MO, USA) at −102 °C and 0.08–0.120 mbar for 2 days.

#### 4.1.6. Characterization of Regenerated Cellulose—Scanning Electron Microscopy (SEM)

Freeze-dried, regenerated cellulose samples were mounted on carbon discs without being subjected to any prior coating. The scanning electron micrographs of the regenerated cellulose samples were recorded by a Hitachi TM-1000 tabletop environmental scanning electron microscope at an accelerating voltage of 15 kV (Hitachi, Tokyo, Japan).

#### 4.1.7. Characterization of Regenerated Cellulose—Fourier-Transform Infrared (FTIR) Spectroscopy

Freeze-dried regenerated cellulose samples and the raw cotton fibers (hot-dried ground cotton fibers) were conditioned in the laboratory at 21 ± 1 °C at 65 ± 2% relative humidity for 2 days prior to the FTIR analysis. The FTIR spectra of regenerated cellulose samples were collected using an FTIR instrument (Spotlight 400, Perkin-Elmer, Waltham, MA, USA) that contained an UATR accessory and a ZnSe–Diamond crystal, as described by Abidi et al. [55]. A background scan of the clean ZnSe–Diamond crystal was obtained before the samples were scanned. A small portion from each sample was kept on the clean crystal. Good contact between the sample and the infrared beam was maintained by applying a constant pressure using the “pressure arm”. The FTIR spectra of regenerated cellulose samples were collected in the mid-IR range from 650 to 4000 cm^−1^ (at a resolution of 4 cm^−1^) with 32 co-added scans. Perkin-Elmer software (Perkin-Elmer Software version 10.5) was used to perform the baseline correction and normalization of each spectrum.

### 4.2. Ionic Liquids

#### 4.2.1. Synthesis of ILs

All the ILs were synthesized at the Department of Chemistry and Biochemistry, Texas Tech University (Table 1).

1-Methylimidazole (Sigma, St. Louis, MO, USA, 99%), 1-bromoethane (Sigma, 99%), 1-bromopropane (Sigma, 98%), 1-bromobutane (Sigma, 98%), 1-bromopentane (Sigma, 98%), 1-bromohexane (Sigma, 99%), 1-bromoheptane (Sigma, 99%), 1-bromooctane (Sigma, 99%), and Amberlite IRA-400 chloride resin (Sigma) were utilized. After synthesizing corresponding imidazolium bromide ILs via anion exchange reactions, acetate ILs were generated [30]. The synthesized ILs were dried in a vacuum oven at 50 °C for 96 h. After drying, the water content was measured by Karl Fisher titration (less than 1000 ppm; Karl Fisher METTLER TOLEDO C20 coulometric KF titrator, Thermo Fisher Scientific, Waltham, MA, USA). Halide tests were performed to detect the presence of halogen ions by using AgNO_3_, and no precipitation was observed (undetectable). The purity of the synthesized ILs was determined using a previously reported protocol (the purity of all synthesized ILs is above 97%) [20]. A JEOL ECS 400 MHz spectrometer was used to record 1H NMR spectra. For the 1H NMR spectra, tetramethyl silane (TMS; δ = 0.00) or the residual protic solvent peak (for DMSO (d6); δ = 2.50) served as a shift reference. Coupling constants, J, are reported in hertz. The following abbreviations were used to describe peak multiplicities in the reported NMR spectroscopic data: “s” for singlet, “d” for doublet, “t” for triplet, “q” for quartet, and “m” for multiplet.

#### 4.2.2. General Synthesis of 1-Alkyl-3-methylimidazolium Acetate Ionic Liquids

1-alkyl-3-methylimidazoliumbromides (alkyl = ethyl, propyl, butyl, pentyl, heptyl, octyl) were synthesized by reacting stoichiometric amounts of corresponding alkyl bromides with 1-methylimidazole. After the isolation and purification of bromide anion-containing ILs, they were dissolved in deionized water, the resulting solutions were passed through an anion exchange column filled with a resin (amberlite IRA-400) to yield aqueous solutions of 1-alkyl-3-methylimidazolium hydroxides, followed by neutralization with acetic acid. An IL/water mixture was placed in a fume hood, and water was evaporated under normal conditions by leaving it in the hood for few days. The residual liquid was repeatedly washed with excess anhydrous diethyl ether. To the resulting liquids dichloromethane and activated carbon were added and stirred for 3 days. Afterward, mixtures were passed through a gravitational column consisting of celite, sand, and alumina. Dichloromethane was removed by rotary evaporation, and the product was dried in a vacuum at room temperature.

^1^H NMR characterization of ILs.

^1^H NMR (400 MHz, DMSO-d_6_) for [C_2_C_1_im][CH_3_COO]: 0.80 (3H, t, CH_3_) 1.50 (3H, s, CH_3_CO_2_), 3.86 (3H, s, NCH_3_), 4.18 (2H, q, NCH_2_), 7.80 (1H, s, NCH), 7.90 (1H, s, NCH), 10.36 (1H, s, NCHN).

^1^H NMR (400 MHz, DMSO-d_6_) for [C_3_C_1_im][CH_3_COO]: 0.82 (3H, t, CH_3_) 1.49 (3H, s, CH_3_CO_2_), 3.84 (3H, s, NCH_3_), 4.24 (2H, m, NCH_2_CH_2_), 7.80 (1H, s, NCH), 7.90 (1H, s, NCH), 10.36 (1H, s, NCHN).

^1^H NMR (400 MHz, DMSO-d_6_) for [C_4_C_1_im][CH_3_COO]: 0.83 (3H, t, but-CH_3_), 1.20 (2H, m, CH_2_), 1.55 (3H, s, CH_3_CO_2_), 1.70 (2H, m, CH_2_), 3.86 (3H, s, NCH_3_), 4.21 (2H, t, NCH_2_), 7.80 (1H, s, NCH), 7.90 (1H, s, NCH), 10.36 (1H, s, NCHN).

^1^H NMR (400 MHz, DMSO-d_6_) for [C_5_C_1_im][CH_3_COO]: ]: 0.84 (t, *J* = 7.2 Hz, 3H, CH_2_CH_3_), 1.32–1.38 (m, *J* = 7.0 Hz, 8H, CH_2_CH_2_CH_2_CH_2_CH_3_), 1.70 (s, 3H, CH_3_C=O), 4.12 (s, 3H, N-CH_3_), 4.47 (d, 2H, *J* = 7.0 Hz, N-CH_2_-), 8.09 (d, 2H, *J* = 12.0 Hz, NCHCHN), 10.68 (s, 1H, NCHN) ppm.

^1^H NMR (400 MHz, DMSO-d_6_) for [C_6_C_1_im][CH_3_COO]: 0.86 (t, *J* = 7.2 Hz, 3H, CH_2_CH_3_), 1.32–1.38 (m, *J* = 7.0 Hz, 10H, CH_2_CH_2_CH_2_CH_2_CH_2_CH_3_), 1.70 (s, 3H, CH_3_C=O), 4.12 (s, 3H, N-CH_3_), 4.47 (d, 2H, *J* = 7.0 Hz, N-CH_2_-), 8.09 (d, 2H, *J* = 12.0 Hz, NCHCHN), 10.68 (s, 1H, NCHN) ppm.

^1^H NMR (400 MHz, DMSO-d_6_) for [C_7_C_1_im][CH_3_COO]: 0.88 (t, *J* = 7.2 Hz, 3H, CH_2_CH_3_), 1.30–1.42 (m, *J* = 7.0 Hz, 12H, CH_2_CH_2_CH_2_CH_2_CH_2_CH_2_CH_3_), 1.75 (s, 3H, CH_3_C=O),4.16 (s, 3H, N-CH_3_), 4.51 (d, 2H, *J* = 7.0 Hz, N-CH_2_-), 8.12 (d, 2H, *J* = 12.0 Hz, NCHCHN), 10.72 (s, 1H, NCHN) ppm.

^1^H NMR (400 MHz, DMSO-d_6_) for [C_8_C_1_im][CH_3_COO]: 0.91 (t, *J* = 7.2 Hz, 3H, CH_2_CH_3_), 1.28–1.44 (m, *J* = 7.0 Hz, 14H, CH_2_CH_2_CH_2_CH_2_CH_2_CH_2_CH_2_CH_3_), 1.82 (s, 3H, CH_3_C=O), 4.22 (s, 3H, N-CH_3_), 4.56 (d, 2H, *J* = 7.0 Hz, N-CH_2_-), 8.22 (d, 2H, *J* = 12.0 Hz, NCHCHN), 10.80 (s, 1H, NCHN) ppm.

## 5. Conclusions

The effect of the alkyl chain length of imidazolium-based acetate ILs on the dissolution of cellulose was investigated. The ILs with chain lengths ranging from C2 to C6 completely dissolved 5% (*w*/*w*) cotton cellulose within 24 h at 90 °C. Among these ILs, [C_2_C_1_im][OAc], [C_3_C_1_im][OAc], and [C_4_C_1_im][OAc] were even more effective in the dissolution of cotton cellulose at 90 °C. The ILs containing long alkyl chains, i.e., [C_7_C_1_im][OAc] and [C_8_C_1_im][OAc], were less effective, and complete dissolution was not achieved even after 24 h. However, in IL [C_7_C_1_im][OAc], swollen cotton fibers were noticed. The SEM images indicated that the cellulose regenerated from [C_2_C_1_im][OAc], [C_3_C_1_im][OAc], [C_4_C_1_im][OAc], [C_5_C_1_im][OAc], and [C_6_C_1_im][OAc] formed uniform structures, whereas the cellulose regenerated from [C_7_C_1_im][OAc] and [C_8_C_1_im][OAc] mainly consisted of undissolved cotton fibers. The FTIR spectra of cellulose regenerated from solutions in ILs with carbon chains from C2 to C6 have a cellulose II structure, whereas those of regenerated cellulose obtained from the least efficient ILs ([C_7_C_1_im][OAc] and [C_8_C_1_im][OAc]) have a cellulose I structure. The odd–even effect of alkyl chain lengths is supported by this study, which found that even C2- and C4-containing ILs were more effective as cellulose solvents than odd C3- and C5-containing ILs. However, such a distinct behavior was not observed in C6-to-C8 alkyl chain-containing ILs. Cotton was used as the cellulose source in this study, which ultimately made it possible to generate cellulose-based biomaterials.

## Figures and Tables

**Table 1:** The synthesized ILs and their abbreviations.

No. of Carbons in Alkyl Chain	Name of IL	Abbreviation
2	1-ethyl-3-methylimidazolium acetate	[C_2_C_1_im][OAc]
3	1-propyl-3-methylimidazolium acetate	[C_3_C_1_im][OAc]
4	1-butyl-3-methylimidazolium acetate	[C_4_C_1_im][OAc]
5	1-pentyl-3-methylimidazolium acetate	[C_5_C_1_im][OAc]
6	1-hexyl-3-methylimidazolium acetate	[C_6_C_1_im][OAc]
7	1-heptyl-3-methylimidazolium acetate	[C_7_C_1_im][OAc]
8	1-octyl-3-methylimidazolium acetate	[C_8_C_1_im][OAc]

## Data Availability

The original contributions presented in this study are included in the article/Supplementary Material. Further inquiries can be directed to the corresponding author(s).

## Supplementary Materials

The following supporting information can be downloaded at: https://www.mdpi.com/article/10.3390/molecules30132711/s1, Figure S1: PLM images of cotton dissolved in (**a**) [C2C1im][OAc], (**b**) [C3C1im][OAc], and (**c**) [C4C1im][OAc] for 8 h at 90 °C; Figure S2: FTIR spectra of hot-dried ground cotton fibers and cellulose regenerated after dissolving in (**a**) [C_3_C_1_im][OAc], (**b**) [C_4_C_1_im][OAc], (**c**) [C_5_C_1_im][OAc], (**d**) [C_6_C_1_im][OAc], and (**e**) [C_7_C_1_im][OAc].
