# Supplementary material for: Effect of Alkyl Chain Length on Dissolution and Regeneration Behavior of Cotton in 1-Alkyl-3-methylimidazolium Acetate Ionic Liquids
Source: Molecules. 2025 Jun 24;30(13):2711. doi: 10.3390/molecules30132711 (PMC12250992; doi:10.3390/molecules30132711)
Supplement: Supplementary file 1 [file molecules-30-02711-s001.zip › molecules-3680306-supplementary.pdf]

Effect of Alkyl Chain Length on the Dissolution Behavior of Cotton in 1-Alkyl-3-methylimidazolium Acetate Ionic Liquids

Supplementary Materials:

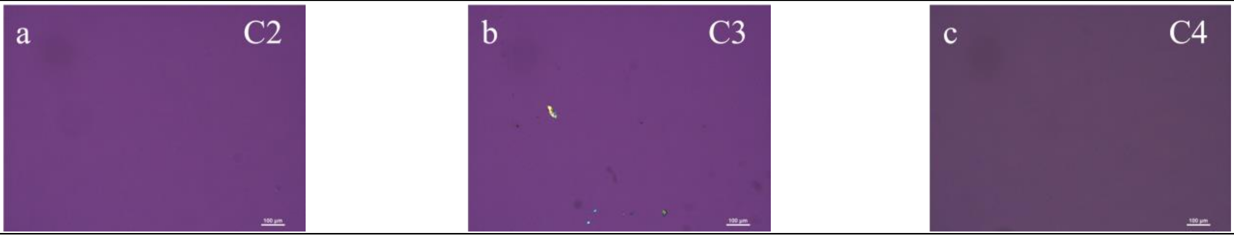

Figure S1: PLM images of cotton dissolved in: (**a**) $[C_2C_1im][OAc]$, (**b**) $[C_3C_1im][OAc]$, and (**c**) $[C_4C_1im][OAc]$ 8 hrs at 90 °C

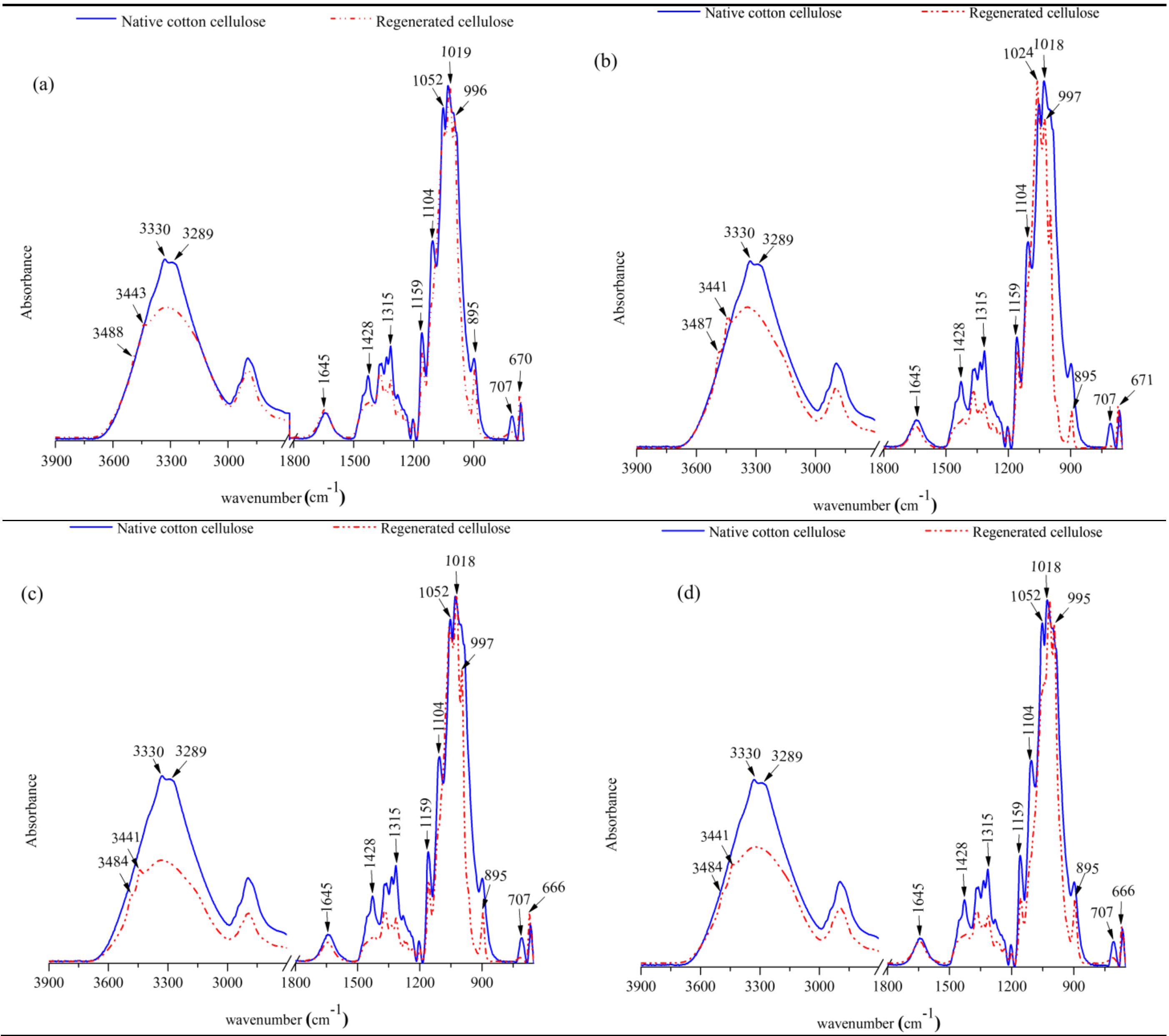

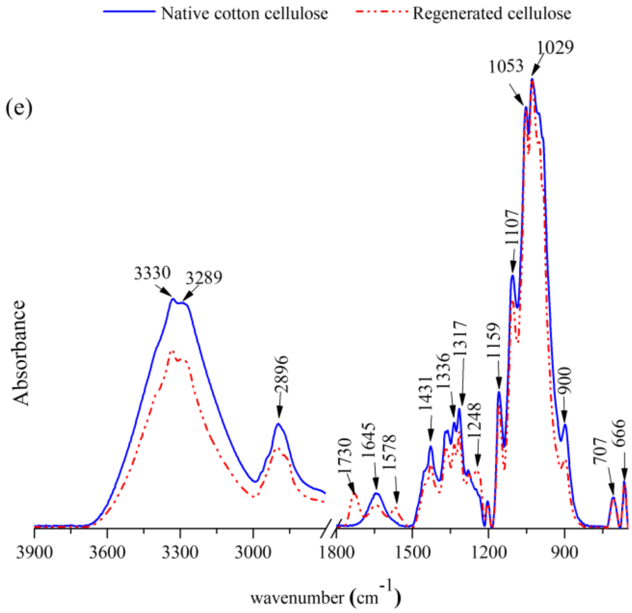

Figure S2: FTIR spectra of hot-dried ground cotton fibers and cellulose regenerated after dissolving in (**a**) [C_3C_1im][OAc], (**b**) [C_4C_1im][OAc], (**c**) [C_5C_1im][OAc], (**d**) [C_6C_1im][OAc], and (**e**) [C_7C_1im][OAc].
